# Neutralization of IL-33 modifies the type 2 and type 3 inflammatory signature of viral induced asthma exacerbation

**DOI:** 10.1186/s12931-021-01799-5

**Published:** 2021-07-15

**Authors:** Kristi J. Warren, Jill A. Poole, Jenea M. Sweeter, Jane M. DeVasure, John D. Dickinson, R. Stokes Peebles, Todd A. Wyatt

**Affiliations:** 1 Critical Care and Sleep Division, Department of Internal Medicine, Pulmonary, University of Nebraska Medical Center, Omaha, USA; 2 Department of Environmental, Agricultural and Occupational Health, University of Nebraska Medical Center, Omaha, NE 68198-5910 USA; 3 Division of Allergy, Pulmonary and Critical Care Medicine, Department of Medicine, Vanderbilt University, Nashville, USA; 4 VA Nebraska-Western Iowa Health Care System, Omaha, NE 68105 USA; 5 Division of Pulmonary Medicine, Department of Internal Medicine, University of Utah Health, 26 N 1900 E, Salt Lake City, UT 84132 USA; 6 VA Salt Lake City Health Care System, Salt Lake City, UT 84148 USA

**Keywords:** Interleukin-33 (IL-33), Interleukin-23 (IL-23), Thymic stromal lymphopoietin (TSLP), Group 2 innate lymphoid cells (ILC2), Group 3 innate lymphoid cells (ILC3), Respiratory syncytial virus-A2 (RSV-A2)

## Abstract

**Background:**

Respiratory viral infections are one of the leading causes of need for emergency care and hospitalizations in asthmatic individuals, and airway-secreted cytokines are released within hours of viral infection to initiate these exacerbations. IL-33, specifically, contributes to these allergic exacerbations by amplifying type 2 inflammation. We hypothesized that blocking IL-33 in RSV-induced exacerbation would significantly reduce allergic inflammation.

**Methods:**

Sensitized BALB/c mice were challenged with aerosolized ovalbumin (OVA) to establish allergic inflammation, followed by RSV-A2 infection to yield four treatment groups: saline only (Saline), RSV-infected alone (RSV), OVA alone (OVA), and OVA-treated with RSV infection (OVA-RSV). Lung outcomes included lung mRNA and protein markers of allergic inflammation, histology for mucus cell metaplasia and lung immune cell influx by cytospin and flow cytometry.

**Results:**

While thymic stromal lymphopoietin (TSLP) and IL-33 were detected 6 h after RSV infection in the OVA-RSV mice, IL-23 protein was uniquely upregulated in RSV-infected mice alone. OVA-RSV animals varied from RSV- or OVA-treated mice as they had increased lung eosinophils, neutrophils, group 2 innate lymphoid cells (ILC2) and group 3 innate lymphoid cells (ILC3) detectable as early as 6 h after RSV infection. Neutralized IL-33 significantly reduced ILC2 and eosinophils, and the prototypical allergic proteins, IL-5, IL-13, CCL17 and CCL22 in OVA-RSV mice. Numbers of neutrophils and ILC3 were also reduced with anti-IL-33 treatment in both RSV and OVA-RSV treated animals as well.

**Conclusions:**

Taken together, our findings indicate a broad reduction in allergic-proinflammatory events mediated by IL-33 neutralization in RSV-induced asthma exacerbation.

## Background

Asthma is a chronic airway disease characterized by type 2-mediated inflammation that promotes mucus cell metaplasia and airway hyperresponsiveness (AHR). Respiratory viral infections commonly exacerbate and, thereby, worsen asthmatic airway disease [1–3]. Viruses trigger asthma exacerbations by damaging or necrotizing airway cells leading to the release of airway epithelial cell-derived cytokines, like thymic stromal lymphopoietin (TSLP), IL-25 and IL-33. These cytokines go on to perpetuate the type 2 inflammatory lung profile by activating resident immune populations such as group 2 innate lymphoid cells (ILC2) and IL-13 polarized (M2) macrophages [4–7].

As respiratory viral pathogens are often detected in humans following asthma exacerbations [4, 8, 9], animal models are used to study the specific contributions of respiratory viruses that trigger inflammation in experimental asthma [10–12]. In stand-alone respiratory infections, interferon-gamma (IFNg) producing T helper (TH1) cell responses are necessary to clear intracellular pathogens, however, the type 3 immune signature, mediated by IL-17 producing T helper (TH17) cells, is amplified in the combination of allergic and respiratory viral challenge [13]. With the more recent identification of group 2 and group 3 innate lymphoid cell subsets, or ILC2 and ILC3, that mimic IL-13 producing T helper (TH2) and TH17 cells in cytokine production, respectively, we chose to examine these innate lymphoid cell populations for their contributions to viral-induced asthma exacerbation. We further chose Respiratory Syncytial Virus-A2 (RSV-A2) for our studies as the RSV-specific response generates substantial TSLP, IL-25 and IL-33 and mucus production [6, 7, 14], and this virus has been well characterized for exacerbating OVA-induced inflammation previously [15–20]. Clinical data also suggests that asthmatic individuals are at greater risk of respiratory complication from community-acquired RSV infections in comparison to otherwise healthy counterparts [18, 21, 22].

IL-33 is an innate cytokine constitutively expressed in airway epithelial cells and several reports detect IL-33 release from the airway epithelium following cigarette smoke exposure, protease treatment, influenza A viral infection, rhinovirus infection, house dust mite exposure, cockroach antigen stimulation and *Alternaria* extract exposure [23–26]. IL-33 can also be induced in mast cells and macrophages under allergic conditions and RSV infection [14, 27]. Moreover, in ovalbumin (OVA)-induced allergic inflammation, anti-IL-33 treatment significantly reduces mucus production in small airways. In studies involving non-allergic lung insult, inhibiting the IL-33 signaling pathway results in reduced immunopathology following RSV infection [28–30]. Based upon these observations, we hypothesized that IL-33 is important to mediating RSV-exacerbated allergic inflammation by directly increasing ILC2. Herein, we show unique features of RSV, OVA and OVA-RSV induced allergic inflammation that are regulated by IL-33. In summary, markers of allergic inflammation, mucus production, lung eosinophils and ILC2 are regulated by IL-33 expression following OVA-challenge and RSV infection.

## Methods

### Animal model

**Fig. 1:**
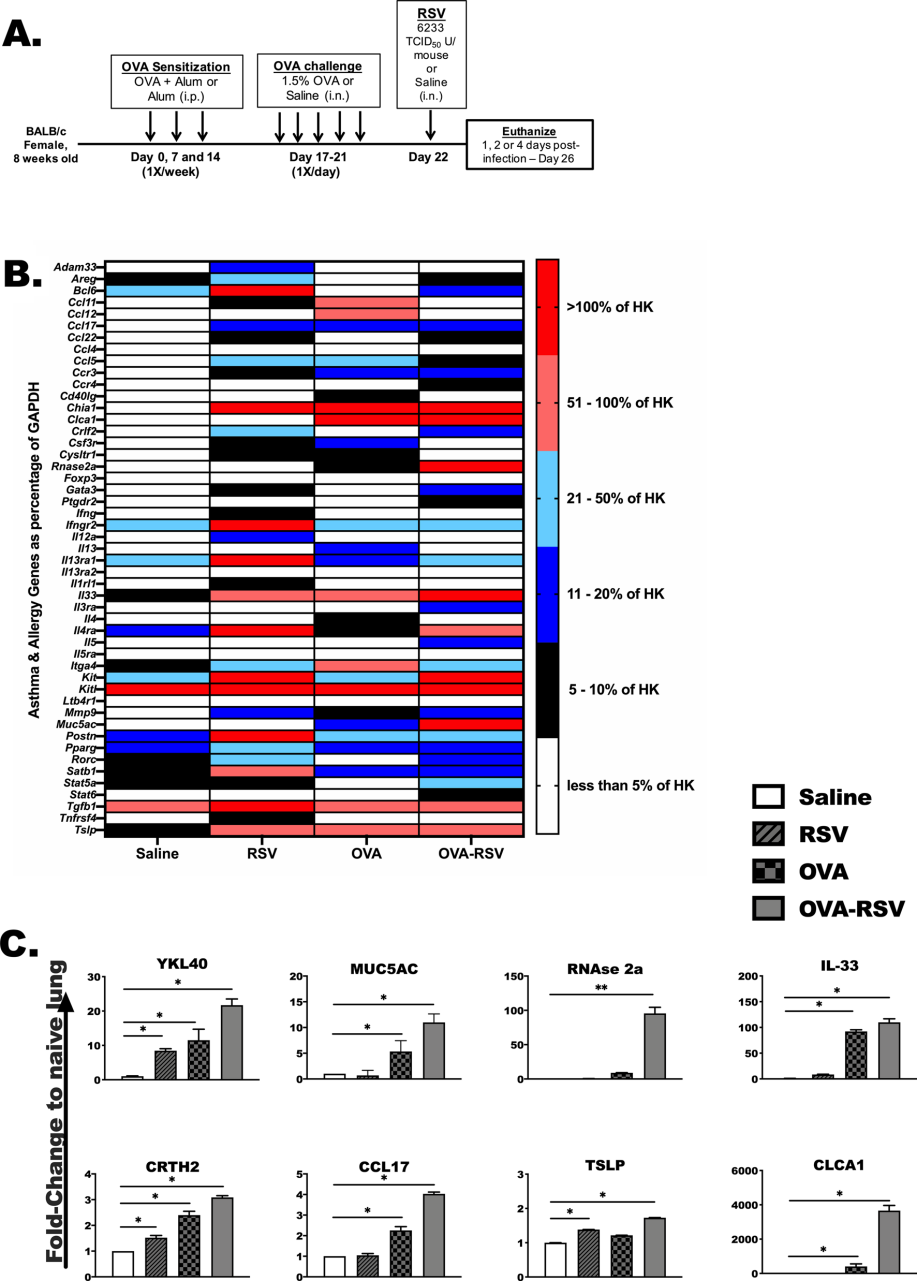
Biomarkers of allergic lung inflammation are augmented in allergic-OVA mice infected with RSV. **A** Mice were intranasally infected with purified RSV-A2 24 h following final OVA airway challenge. Four groups of mice were included in each experiment; saline-treated alone (Saline), RSV-infected (RSV), OVA-treated alone (OVA), and OVA-treated, RSV-infected (OVA-RSV). **B**, **C** Total lung RNA was examined for RNA expression of common allergic markers using a RT^2^ PCR array. For all experiments, N = 3–4 mice/group. ANOVA was used to determine of statistical significance and Kruskal–Wallis post-test analysis was used to determine the statistical significance between groups. *Indicates *P* < 0.05

Eight-week old, female, BALB/c mice (Charles River, O’Fallon, MO) were acclimated at our facility for at least one week prior to experimental procedures. All mice had ad libitum access to standard rodent chow and filtered water. Mice were allergen-sensitized with 100 μL of chicken egg ovalbumin (Grade V; 500 μg/mL) adsorbed with aluminum hydroxide (Sigma, 20 mg/mL), or mice were mock sensitized with an intraperitoneal (i.p.) injection of aluminum hydroxide alone (Alum), on days 0, 4 and 7. To induce allergic airway inflammation, mice underwent airway challenge with 1.5% OVA in saline (designated OVA group) or saline alone for 40 min per day (day 17–21) in a whole-body ultrasonic nebulization chamber (DeVilbiss). A subset of mice from the OVA and saline alone groups were infected on day 22 with purified RSV-A2 (6233 TCID_50_ U/mouse, Advanced Biotechnologies, Columbia, MD) diluted in sterile saline (OVA-RSV group and RSV group, respectively), or saline alone (saline and OVA groups) (Fig. 1A). For the IL-33 neutralization experiments, mice were given an i.p. injection of anti-IL-33 monoclonal antibody or an irrelevant IgG control antibody of the same isotype at a dose of 6 μg per mouse diluted in 100 μL of saline (BioXcell, West Lebanon, NH) on days 21 and 25 of experimental procedure (Fig. 6A). Subsets of mice from each group were euthanized on days 22, 24 and 26 of experimental procedure, or day 1 (6 h), day 2 or day 4 post-RSV infection, for bronchoalveolar lavage fluid (BALF) collection, lung perfusion and tissue collection. All animal procedures were conducted under the approval of the University of Nebraska Medical Center Institutional Animal Care and Use Committee (IACUC).

### Lung histopathology

The left lungs were inflated with 0.8 ml of 10% formalin and hung under a pressure of 20 cm^2^ for 24 h while submerged in 10% formalin solution to obtain uniform lung inflammation during fixation. Lung tissue was subsequently embedded and stained by H & E (hematoxylin & eosin) and Periodic-Acid Schiff (PAS) for mucin determination by standard staining procedures. A scoring method was developed to quantify the degree of PAS + staining in the moderate to large airways. The slides were scored in a blinded fashion by determining the total number of PAS + stained epithelial cells divided by the number of total airway epithelial cells in an airway. The airways were evaluated in 8–10 airways/section, 3 consecutive 8 micron sections/slide, two slides/animal, and 3–5 animals/group. The percent of airway epithelial cells that were PAS + was determined by dividing the total number of PAS + epithelial cells by the total number of epithelial cells multiplied by 100. Next, the percentage was assigned an arbitrary score for each airway: 0 = 0–25% PAS + , 1 = 25–50% PAS + , 2 = 50–75% and 3 = 75–100%; or displayed as percentage of PAS + airway epithelial cells. The mean cumulative score for each group was determined and evaluated by statistical method for significance.

### Flow cytometry

Bronchoalveolar Lavage fluid (BALF) was collected as previously described [31], and BALF cells were separated from supernatants by centrifugation immediately after collection. Cells were counted and suspended in phosphate buffered saline (PBS; pH 7.4) containing 2% fetal bovine serum (FBS) and 0.01% sodium azide. Cells were isolated from remaining right lung tissue as previously described [32]. Briefly, following euthanasia and lung lavage, the right ventricle was infused with 10 mL sterile PBS to remove blood from the pulmonary vasculature. Lungs were dissociated by pressing through a 40 µm nylon cell strainer (Thermo Fisher Scientific, Waltham, MA) into a Petri dish; cell dissociation was carried out in a solution containing collagenase type I (324 U/mL; Fisher, Pittsburgh, PA). After dissociation, cell suspensions were incubated at 37 °C for 15 min to activate the collagenase. The resulting suspension was passed through a second 40-micron nylon mesh strainer to remove remaining large fragments. Cells were washed one time by centrifugation (378×*g*) and re-suspended in Hank’s balanced salt solution (HBSS) without Ca + or Mg + . Cell suspensions were gently layered onto a Ficoll gradient and centrifuged with no brake for 35 min at 300×*g*. Final cell counts for BAL cells and total lung leukocytes were obtained using a hemocytometer. Viability of the final cell preparations was assessed at greater than 95% viable by trypan blue exclusion. Cell suspensions from each animal were incubated with anti-CD16/32 (F_c_ Block, BD Biosciences, San Jose, CA) to minimize non-specific antibody staining and stained with anti-mouse antibodies against CD3, CD19, CD11c, CD11b, CD45, NK1.1, F4/80, Ly6g, Ly6c, Sca-1, Siglec-F, ST2, KLRG-1 and ICOS (BD Biosciences, San Jose, CA). Parallel cell preparations were treated with appropriate isotype controls. Cytometer compensation was performed with antibody capture beads (eBioscience, Grand Island, NY) stained separately with individual antibodies used in test samples.

All populations were gated by characteristic forward and side-scatter properties and antibody-specific staining fluorescence intensity using a FACS Aria cell sorter system and associated software (BD Biosciences). The gating strategy for CD45 + total T lymphocytes (CD45 + CD3 +), B lymphocytes (CD45 + CD3-CD19 +), as previously published [31]. Eosinophils were quantitated at SSC + CD11c-CD11b + Siglec-F + cells. Group 2 innate lymphoid cells (ILC2) were lineage negative (LIN-) CD3-CD19-CD11b-CD11c-NK1.1-F4/80-NKp46- followed by inclusive gating on ST2 and ICOS. To determine the numbers of each cell population, initial gating on CD45^+^ lung leukocytes excluded debris, and the percentage of respective populations was determined from total cells. This cytometric percentage was multiplied by the original hemocytometer count of total cells recovered for each animal to provide the total number of each cell type from the BAL fluid or whole lung tissue.

### Lung lymphoid cell isolation and activation

Lung lymphocytes were isolated from homogenized lung tissue for the intracellular staining experiments using the method described previously [33]. For the IL-33 and IL-23 cell culture experiments, lung ILC2 sorting was conducted as previously published [34], and pan innate lymphoid cells were enriched using StemCell pan ILC enrichment kit (Tempe, AZ). Briefly, lung tissue was dissociated, processed, and labeled with antibodies to common immune lineage markers, as described in the previous section. Gating was set on a FACS Aria (BD Biosciences) to acquire CD45 + lineage negative (LIN-) ST2 + ICOS + group 2 innate lymphoid cells. Immediately following sorting, cells were washed one time with sorting buffer (PBS, 0.5% BSA) and between 1.5 and 2.5 × 10^3^ ILC2 were seeded into culture wells in a 96-well U-bottom plate (Falcon, Corning, NY). After 24 h, ILC2 were stimulated with IL-2 (10 ng/ml), IL-7 (10 ng/mL), TSLP (10 ng/mL), IL-25 (10 ng/mL) and ± IL-33 (10 ng/mL). Enriched pan ILC were seeded at a rate of 20,000 cells per well and stimulated with IL-2, IL-7, IL-23 (10 ng/mL) and IL-33 at previously defined amounts. After 72 h, cells were separated from supernatants by centrifugation at 378×*g* for 10 min. Supernatants were stored at − 80 °C until analysis by ELISA.

### RNA isolation and quantitative RT-PCR

RNA was isolated from perfused lung tissue by homogenizing lung tissue in RLT tissue lysis buffer following the instructions provided in the Qiagen RNeasy Mini Kit. 1000 ng of total lung RNA was assayed through the RT^2^ First Strand Synthesis kit (Qiagen, Germantown, MD) to eliminate genomic DNA contamination and synthesize purified cDNA. The total product of this reaction was diluted in SYBR Green ROX qPCR master mix and nuclease-free, deionized and distilled water (Hyclone, Logan, UT) and distributed across a 96-well Mouse Allergy & Asthma RT^2^ Profiler PCR array (Qiagen). The plate was analyzed on the ABI 7500 using a 25 uL reaction volume and cycling at 95.0 °C for 10 min, followed by 40 cycles at 95.0 °C for 15 s, 60.0 °C for 1 min. Ct values below 35 cycles were considered positive, and analysis for changes in gene expression was completed using the data analysis software available from Qiagen. RSV, OVA and OVA-RSV treated lung were compared to saline-treated lungs as baseline for the fold-change.

### ELISA

BALF was centrifuged to remove cells and debris as previously described [31], and supernatants were stored at − 80 °C until cytokines and chemokines were evaluated by ELISA. Cell culture supernatants were precleared by centrifugation (500×*g*, 10 min) and diluted 10- to 100-fold in RPMI prior to ELISA. BAL supernatants were evaluated for IL-5 and IL-13 using the Ready-SET-go ELISA kits commercially available from Affymetrix, Inc. The IL-5, and IL-13 ELISA kits have a lower limit of detection of 4.0 pg/mL. IL-33 and TSLP were measured by ELISA (R&D Systems); these kits have a lower limit of detection of 13 pg/mL and 4 pg/mL, respectively. CCL17 and CCL22 levels were quantitated using the Quantikine ELISA from R & D Systems (Minneapolis, MN) with lower limit of assay detection at 7.8 pg/mL. IL-23, IL-17 and IL-22 were performed using the R&D Systems Duoset ELISA with a lower limit of detection of 7.8 pg/mL. All ELISAs were conducted according to manufacturer’s instructions.

### Statistical analysis

One-way ANOVA was used to determine statistical differences among groups; between groups comparisons were made using the Kruskal–Wallis multiple comparisons method. A p-value < 0.05 was considered significant. In the PCR array experiments, the statistical analysis software was used according to the manufacturer’s instructions (www.sabiosciences.com). In all other experiments, statistical analyses were performed using the statistics software incorporated into GraphPad Prism, Version 10 (La Jolla, CA).

## Results

### Biomarkers specific to allergic lung inflammation are augmented in allergic-OVA mice infected with RSV

Female BALB/c mice were sensitized and challenged with ovalbumin (OVA) followed by intranasal infection with RSV-2A (Fig. 1A). At day 26 of the experimental protocol, allergy-associated gene expression was determined in whole lung tissue by RT-PCR array (Fig. 1B). We hypothesized that the combination of OVA treatment and RSV infection would induce a unique allergic-inflammatory gene profile in comparison to RSV and OVA treatment alone. RSV infection increased (*P* < 0.05) a number of genes as a percentage of our housekeeping gene (*Gapdh*) in comparison to saline-treated lungs; those genes include, *Adam33*, *Areg*, *Bcl6*, *Ccl22*, *Ccr4*, *Cysltr1*, *Crlf2* (TSLP-R), *Ifng*, *Ifngr2*, *Il12a*, *Il1rl1* (IL-33R, or ST2), *Il33*, *Kit*, *Kitl*, *Ltb4r1*, *Mmp9*, *Postn*, *Rorc*, *Satb1*, *Stat6*, *Tnfrsf4* and *Tslp*. As expected, several genes were upregulated in response to OVA sensitization and challenge in comparison to saline-treated animals including *Ccl11, Ccl12, Ccl17, Ccl5, Ccr3, Cd40l, Cysltr1, Il13, Il13ra, Il33, Il4, Itga4,* and *Tslp*. The combination of OVA challenge and RSV infection (OVA-RSV), however, induced *Ccl22*, *Ccr3*, *Ccr4*, *Ccl17*, *Clca1*, *Crlf2*, *Rnase2a*, *Foxp3*, *Gata3*, *Ptgdr1 (CRTH2)*, *Il33*, *Il3ra*, *Il5*, *Kit*, *Mmp9*, *Muc5ac*, *Stat5a*, *Stat6*, and *Tslp* in comparison to saline treated. Several genes were uniquely upregulated in the lungs of OVA-RSV mice in comparison to the OVA mice. Those genes that had significantly (*P* < 0.05) higher expression in the OVA-RSV mice compared to the OVA mice included, *Ccl17*, *Ccl22*, *Ccr4*, *Clca1*, *Crlf2 (*TSLP receptor*)*, *Rnase2a*, *Gata3*, *Ptgdr2*, *Il33*, *Kit*, *Mmp9*, *Muc5ac*, *Rorc*, *Stat5a* and *Stat6* (Fig. 1C). *Tslp* (*P* = 0.08) and the *IL-5Ra* (*P* = 0.12) were slightly elevated in OVA-RSV compared to OVA, but this did not reach statistical significance. *Ccl11*, *Ccl12*, *Ccl5*, *Cd40lg*, *IL-4*, and *Itga4*, interestingly, were suppressed in OVA-RSV in comparison to OVA. These data highlight an important gene expression signature unique to each treatment group.

### IL-33 is rapidly upregulated following allergen challenge and RSV infection

**Fig. 2:**
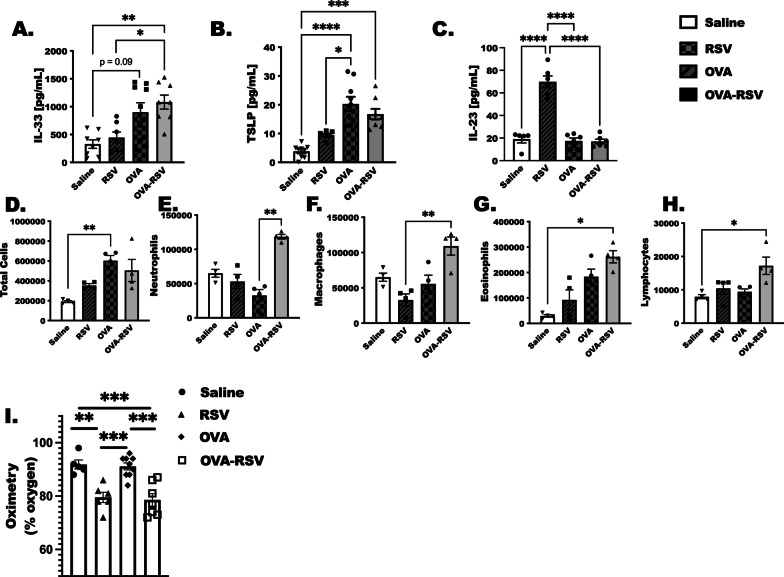
IL-33 is rapidly upregulated following allergen challenge and RSV infection. Six hours after RSV infection mice were euthanized for BAL fluid to assess cytokines by ELISA, and cell populations in BAL cytospins. **A** IL-33, **B** TSLP and **C** IL-23 ELISA were completed in lung homogenates. **D**–**H** BAL cytospins were produced from Saline, RSV, OVA and OVA-RSV treated animals. At least 200 cells were counted per slide, 2 slides per animal and 3–4 animals per group. **I** Pulse oximetry readings were taken 6 h after RSV infection as well. By ANOVA and Kruskal–Wallis post-test. *Indicates *P* < 0.05 differences between groups

Previous studies have examined IL-33, IL-25 and TSLP in respiratory viral infections, lung injury models, and allergen challenge models [6, 7, 35, 36]. Because of these studies, in conjunction with the data presented in Fig. 1, we chose to look at IL-33 and TSLP proteins 6 h after RSV infection in naïve and OVA-challenged mice, comparatively. IL-33 and TSLP were elevated at this time point in the OVA-RSV treated animals in comparison to saline treated animals (Fig. 2A, B). There were no statistical differences between OVA and OVA-RSV treated animals for IL-33 or TSLP, however IL-3 was lower in just RSV infected animals compared to OVA-RSV, and TSLP was lower in RSV compared to OVA alone. Furthermore, because neutrophils and Th17 cells are reported as a means of exacerbating asthma following respiratory infections, we did measure IL-23 protein as well. Surprisingly, only RSV infection induced IL-23 significantly in comparison to all other groups at 6 h after RSV infection (Fig. 2C).

Total leukocytes (Fig. 2D), neutrophils (Fig. 2E), macrophages (Fig. 2F), eosinophils (Fig. 2G), and lymphocytes (Fig. 2H) were quantified in BALF cytospins. As a means of understanding immune cells types, specifically neutrophils and eosinophils, that exacerbate allergic inflammation in humans [37, 38], we show that both populations were significantly elevated in comparison to OVA alone and in comparison to saline, respectively, with the addition of RSV infection after the allergic phenotype is established with OVA. To confirm an effect of RSV infection, we used pulse oximetry to show a reduced blood O_2_ saturation in animals infected with RSV (Fig. 2I). This RSV effect was statistically significant in the OVA-RSV treated animals as well.

### Innate lymphoid cells are increased as early as 6 h after RSV infection in allergic and non-allergic mice

**Fig. 3:**
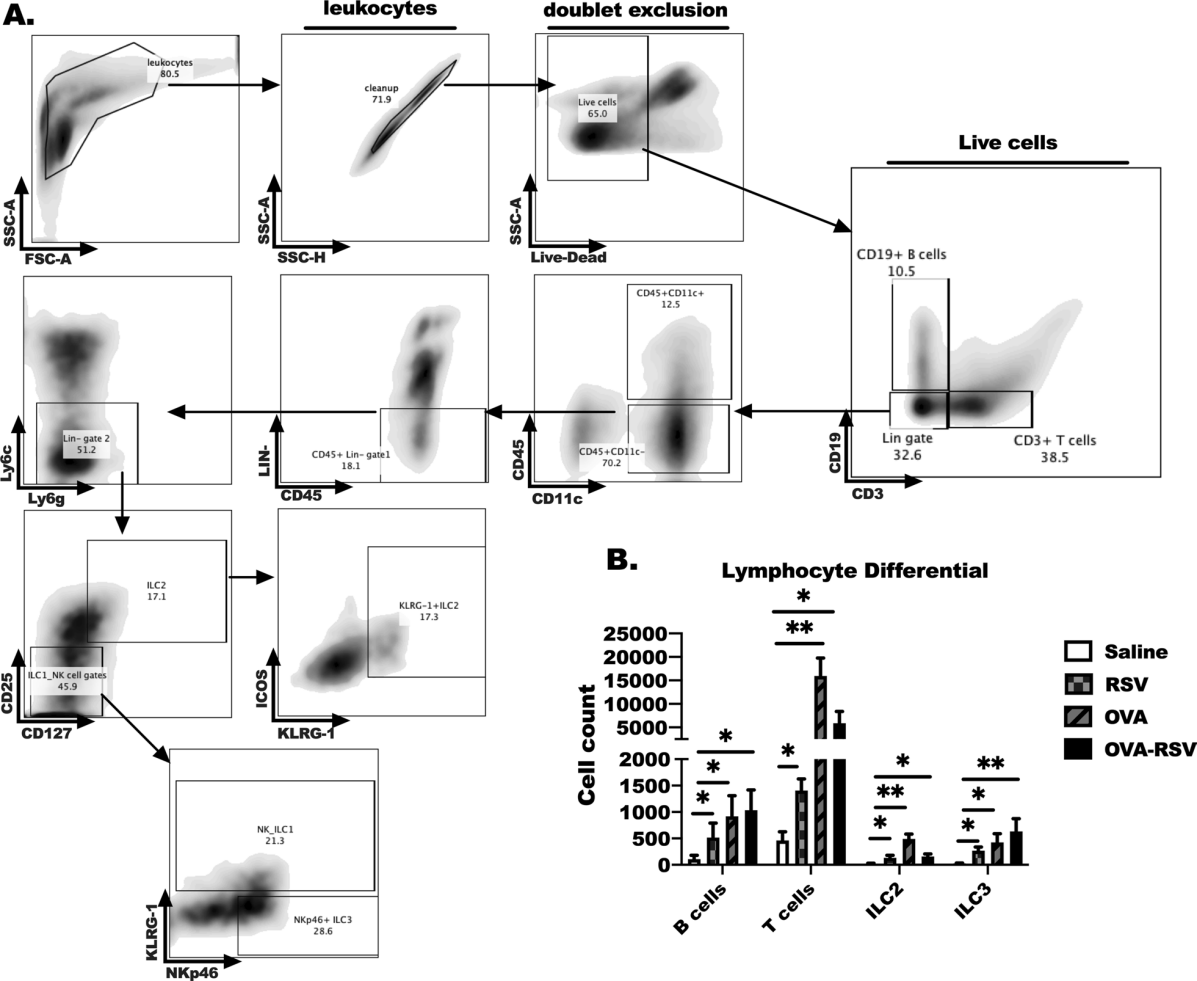
Innate lymphoid cells are increased as early as 6 h after RSV infection in allergic and non-allergic mice. BALF was analyzed by flow cytometry to determine the lymphocyte differential in each of the treatment groups at 6 h after RSV infection. **A** Schematic for flow gating used to determine **B** B cells, T cells, ILC2 and ILC3 are shown. Sample size is 6–9 animals per bar. These results are representative of three independent experiments. Significance (*P < 0.05) was determined using a One-way ANOVA with Kruskal–Wallis post-test

Because lymphocytes were elevated in the BALF cytospins of OVA-RSV treated animals, we completed a lymphocyte differential by flow cytometry that examined multiple lymphoid cell subsets comprehensively in the OVA-RSV infection model. 6 h after RSV infection we detected increased CD19 + B cells, CD3 + T cells, ILC3 and ILC2 in RSV-infected animals compared to saline controls, and both of these populations were elevated in OVA-alone treated animals as well. Nkp46 + ILC3 were also elevated in all groups including RSV, OVA and OVA-RSV treated animals (Fig. 3B). Taken together these data further confirm that each treatment induces a unique host response dependent on the presence or lack of pre-existing allergic inflammation.

### Eosinophils and neutrophils are present at increased numbers in pre-existing allergic airway inflammation that is amplified by RSV infection

**Fig. 4:**
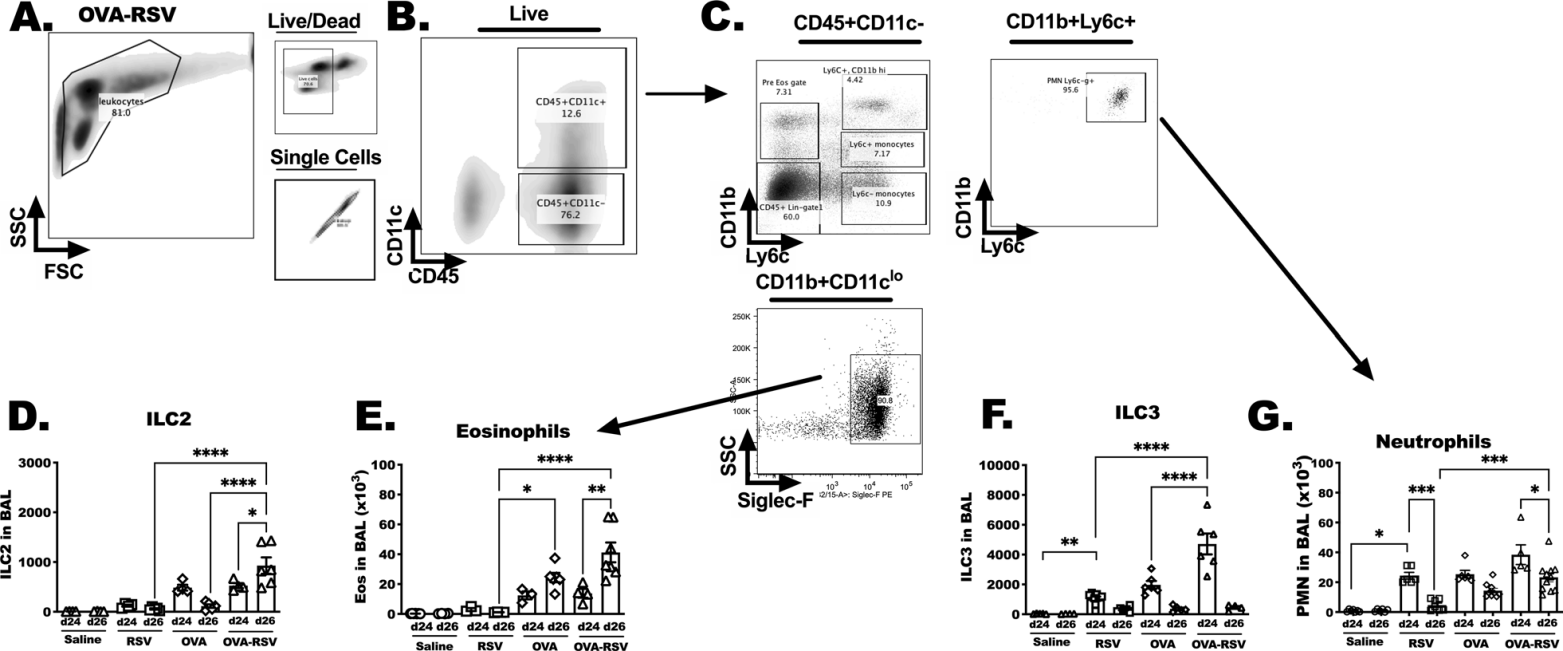
Eosinophils and group 2 innate lymphoid cells are present at increased numbers in pre-existing allergic airway inflammation that is amplified by RSV infection. Eosinophils, neutrophils, group 2 innate lymphoid cells (ILC2) and group 3 innate lymphoid cells were quantitated in BALF at day 24 and 26 by flow cytometry. **A**–**C**, **E** Eosinophils and **A**–**C**, **G** neutrophils were identified as SSC + CD11c-CD11b + Siglec-F + and SSC + CD11c-CD11b + Ly6C + cells, respectively. **D** ILC2 and **F **ILC3 were identified as in Fig. 3. Cell counts were determined using the % total cells acquired in relative gates and multiplied by the total number of cells acquired from each lavage. Data represent five separate experiments (N = 5–6 mice/group for day 2, and N = 10–12 mice/group for day 4). Significance * indicates *P* < 0.05 between treatment groups

Group 3 innate lymphoid cells (ILC3) have not been previously examined in OVA-RSV model, but studies show that IL-23 and TH17 responses are common under similar treatments. Furthermore, neutrophil recruitment to lungs is a well-established complication in respiratory viral infection; neutrophilic responses are supported by IL-23 and IL-17 in lungs. Coincidently, ILC2 activation with IL-33 and TSLP has been shown to support eosinophils and allergic inflammation by subsequent production of IL-5 and IL-13 [7, 29, 39, 40]. Taken together, we hypothesized that group 2 innate lymphoid cells (ILC2) and eosinophils would be increased in the lungs, along with ILC3 and neutrophils at 2 days and 4 days after RSV infection. Indeed, OVA and OVA-RSV-treated mice had significantly more eosinophils at day 24 and day 26 in comparison to saline mice (Fig. 4D, E). However, only OVA-RSV-treated animals had increased eosinophils at day 26 in comparison to OVA-treated animals. The numbers of ILC2 increased from day 24 to 26 in the OVA-RSV mice, and contrastingly, the numbers of ILC2 decreased from day 24 to 26 in OVA mice. Similarly, NKp46 + ILC3 were increased in OVA and OVA-RSV treated mice, however, at day 24, RSV infection induced a marked increase of ILC3 and neutrophils compared to saline treated animals. The numbers of ILC3 and neutrophils were close to baseline levels by day 26 in these studies. These studies show temporal changes in innate immune populations with the most striking data showing that the numbers of ILC2 in the lungs remained higher in the OVA-RSV treated animals in comparison to the OVA-treated alone. The next set of experiments examine whether this was a product of IL-33 expression and secretion.

### IL-33 is a potent stimulator of type 2 cytokine and chemokine release from group 2 innate lymphoid cells

**Fig. 5:**
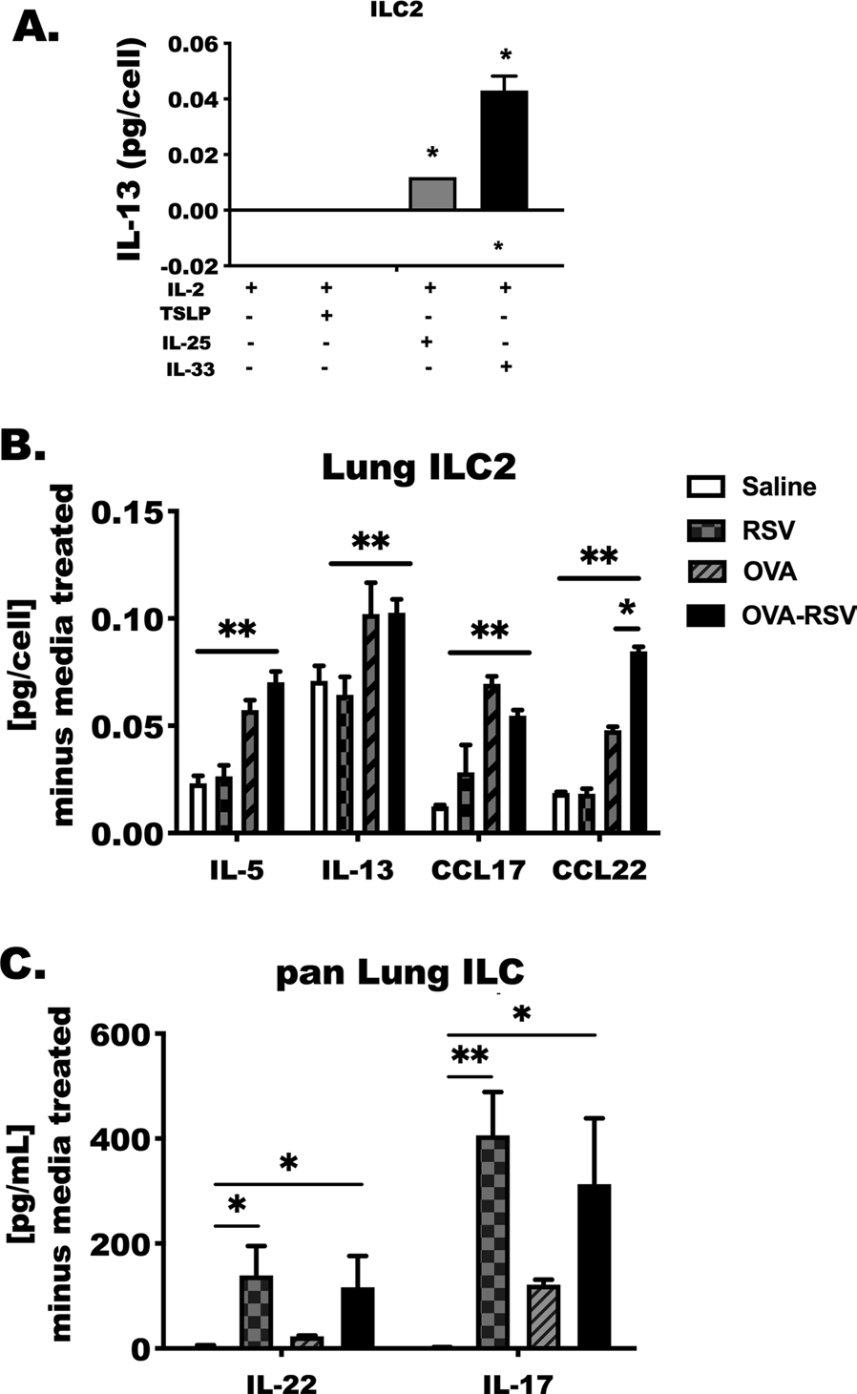
IL-33 is a potent stimulator of type 2 cytokine and chemokine release from group 2 innate lymphoid cells. ILC2 and total ILC were enriched from lung tissue and stimulated with various ILC-activating cytokines. **A** ILC2 from untreated mice were purified using FACS sorting (as in Fig. 3) then stimulated with IL-2 in combination with IL-25, TSLP or IL-33 for 5 days [10 ng/mL, each]. IL-13 was measured in cell culture media by ELISA **B** Lung ILC2 isolated from Saline, RSV, OVA and OVA-RSV treated animals were stimulated with IL-2, IL-7 and IL-33 [10 ng/mL] for 4 days in culture. Cell pellets were counted at the end of 72 h to account for changes in cell divisions or proliferation across groups. IL-5, IL-13, CCL17 and CCL22 levels were assessed by ELISA. Total pg/mL detected in 50 uL of cell culture fluid, was divided by the total number of cells counted in each well at the end of 72 h to determine the pg/cell for each treatment. **C** Total ILC were enriched from the lungs of each subset of animals and stimulated with IL-2, IL-7 and IL-23. IL-22 and IL-17 were measured in cell culture media by ELISA. Each bar represents the mean with standard error of the mean. Cells were pooled from 2 to 3 mice per experiment. Data represent two separate experiments; N = 5–6 samples per bar. *Indicates a statistically significant difference vs. media control (*P* < 0.05). P-value over bar for a difference between groups determined by ANOVA

Because previous studies have identified TSLP, IL-25 and IL-33 as potent activators of innate lymphoid cells in allergic and non-allergic disease states [6, 7, 41], we chose to assess potential activation of lung ILC2 by these epithelium-derived cytokines. Lung ILC2 were isolated from naïve BALB/c animals and cultured with IL-2 in combination with TSLP, IL-25 or IL-33 for 5 days (Fig. 5A). We determined that IL-2 and IL-33 induced a higher IL-13 response as compared to treatment with IL-25 or TSLP, as was similarly shown by Mjosberg et al. [42, 43]. This confirmed that IL-33 is a more potent activator of type 2 inflammatory cytokine by ILC2.

In the next studies we compared the IL-33-induced responsiveness of innate lymphoid cells in separate cultures with the hypothesis that ILC2 from OVA-RSV mice produce more IL-5 and IL-13 than OVA treated animals, and ILC3 produce IL-17 in OVA-RSV mice more readily than ILC3 from OVA-treated animals. Lung ILC2 and pan ILC were enriched from Saline-, RSV-, OVA- and OVA-RSV-treated animals. We found significant increases in IL-33-stimulated IL-13 and IL-5 protein expression in OVA and OVA-RSV mice as compared to saline and RSV alone (*P* < 0.05, Fig. 5B, C). OVA-treated and OVA-RSV treated ILC2 were not different when comparing IL-5 and IL-13 production, however ILC2 from OVA-RSV treated animals produced higher levels of CCL22 on a pg/cell basis in comparison to OVA treated. CCL17 was released from IL-33-stimulated ILC2 from OVA mice to an extent greater than OVA-RSV. There was no difference in IL-5, IL-13 and CCL22 levels between the saline and RSV treatment groups. CCL17 production, however, was significantly increased in IL-33-stimulated ILC2 isolated from RSV-infected mice as compared to saline control. Similar trends were observed with isolated, IL-33-stimulated lung T CD3 + cells (data not shown); however, the magnitude of this response was reduced in comparison to lung ILC2. ILC2 demonstrated an approximate 10- and 15-fold increase in cytokine and chemokine production on a per cell basis when compared to T cells. Total innate lymphoid cells (LIN- cells; or pan ILC) were enriched and stimulated with IL-23 (Fig. 2C) and, importantly, approximately 20,000 of these cells produced IL-22 and IL-17 in RSV and OVA-RSV treated animals only. This was a unique feature of RSV infection that was not seen in OVA or saline treated animals. The IL-23 stimulated pan ILC cultures did not produce detectable levels of IL-5 and IL-13. As we were establishing a role for IL-33 in RSV, OVA and the combination of RSV and OVA challenge we also completed pan ILC culture experiments with both IL-23 and IL-33 co-stimulation. IL-33 had no effect on IL-22 or IL-17 production in the pan ILC experiments.

### Neutralization of systemic IL-33 significantly reduces airway mucus production, and cytokine and chemokine following OVA-RSV treatment

**Fig. 6:**
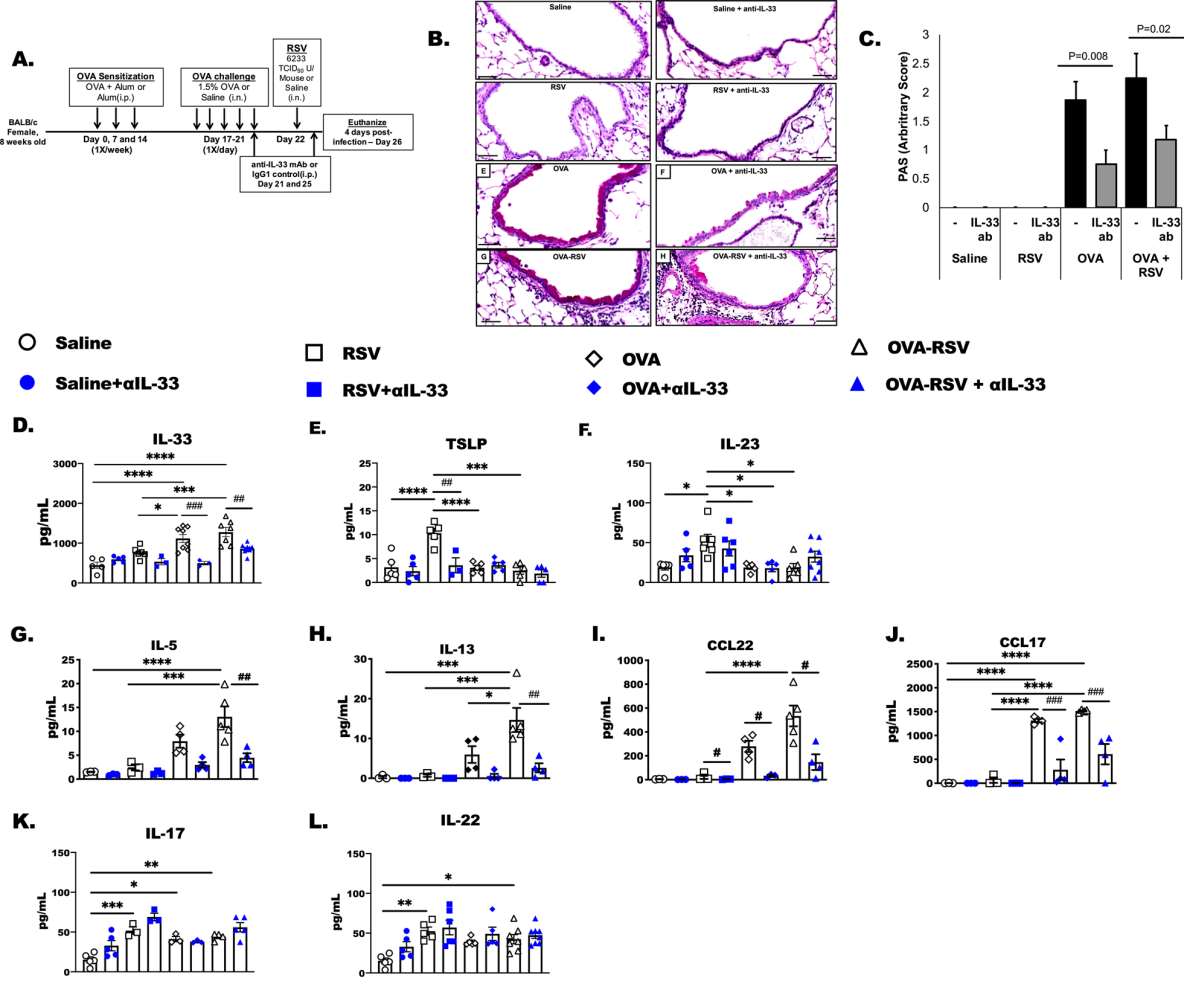
Neutralization of systemic IL-33 significantly reduces airway mucus production, cytokine and chemokine release following OVA and RSV challenge. **A** Female BALB/c mice were treated as before with OVA to induce allergic inflammation. Following the last OVA challenge mice were given and i.p. injection of anti-IL-33 monoclonal antibody (300 ug/kg) followed by RSV infection on day 22. On day 25, anti-IL-33 treated mice were given a second injection of anti-IL-33. **B** Representative lung sections from PAS stained (40× magnification) airways of one mouse from each treatment group. Line scale is 50 μm. **C** depicts the mean with standard error bars of PAS + airway epithelial cell mucin staining. N = 15 airways/groups. **D**–**I** BAL supernates were tested for IL-33, TSLP, IL-23, IL-5, IL-13, CCL22, CCL17, IL-17 and IL-22 proteins by ELISA. Bars represent pg/mL detected per samples ± SEM

We found a significant increase in lung IL-33 mRNA and IL-33 protein in the OVA-RSV group compared to the other groups (Fig. 1C and 2A). We hypothesized that IL-33 was critical for the increased mucus, eosinophilia, and TH2 cytokine production during RSV infection in OVA-allergen challenged mice. To test this hypothesis, we neutralized IL-33 in our model using an anti-IL-33 mAb approach beginning one day prior to RSV infection (Day 21) and again at 3 days after RSV infection (Day 25) (Fig. 6A). Excess mucus production in the airways is a hallmark feature of allergic inflammation and is specifically induced in airway epithelial cells following IL-4 and IL-13 [44, 45]. Here we show that anti-IL-33 treatment significantly decreased mucus production in airway epithelial cells in both OVA and OVA-RSV treated animals (Fig. 6B, C).

In the next studies, the impact of anti-IL-33 mAb on inflammatory cytokines/chemokines was investigated. Anti-IL-33 mAb treatment significantly reduced IL-33 (Fig. 6D), CCL22 (Fig. 6I), CCL17 (Fig. 6J) in the BALF of OVA and OVA-RSV treated animals, and only TSLP was reduced by anti-IL-33 in RSV treated animals (# indicates a significant anti-IL-33 effect; *P* < 0.05). Anti-IL-33 mAb had no effect on IL-23, IL-17 and IL-22 in any of the comparisons, although each one of these cytokines was significantly elevated by RSV infection alone. The anti-IL-33 mAb had no effect on these neutrophil-promoting cytokines at the early, 6 h, time point either, indicating that anti-IL-33 mitigates the type 2 or allergic inflammation, but has no effect on the IL-17 or IL-22 in RSV or OVA-RSV treated animals.

### Unique immune populations arise in RSV, OVA and OVA-RSV treated animals at day 4 after RSV infection

**Fig. 7:**
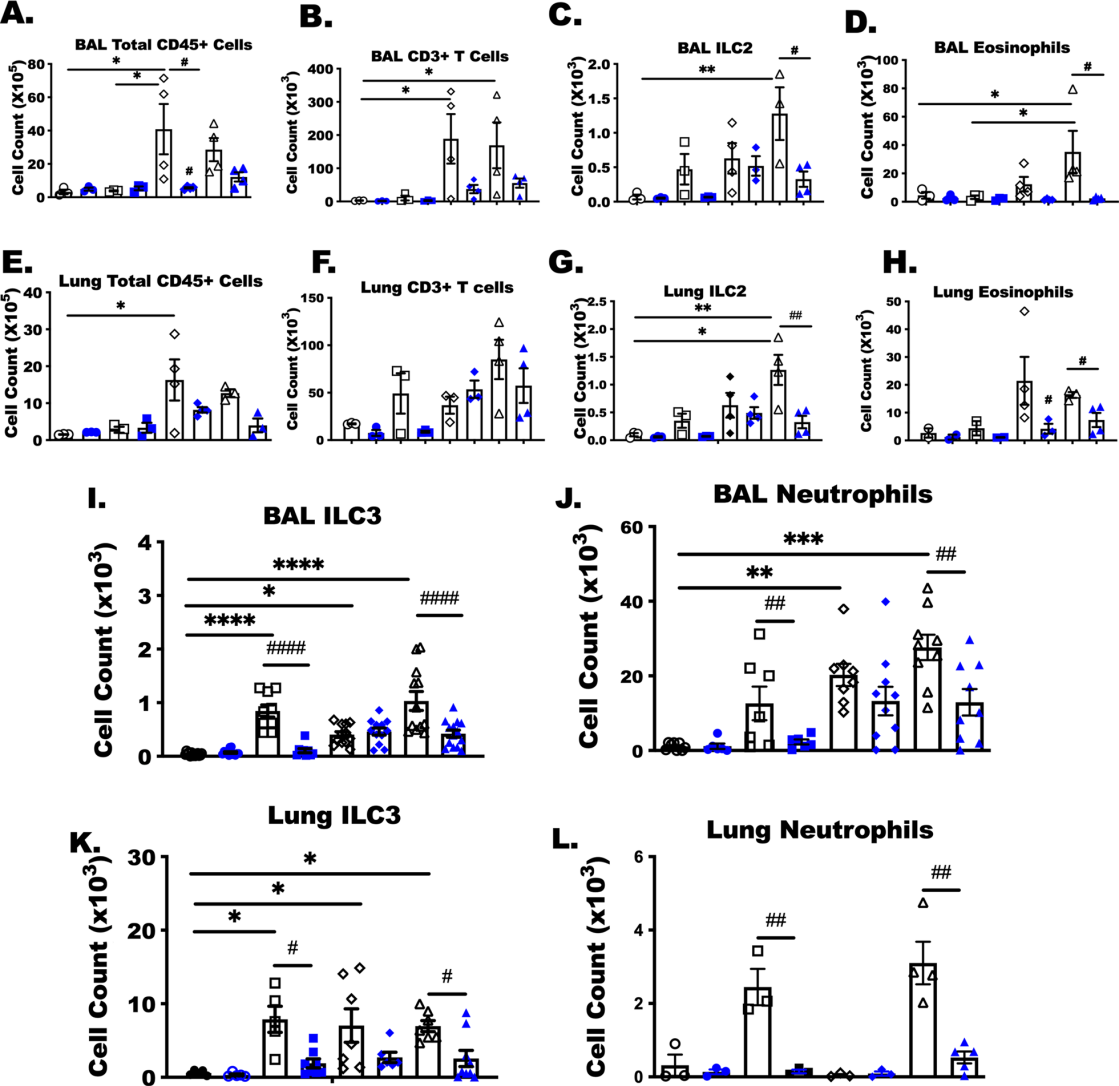
Unique immune populations arise in RSV, OVA and OVA-RSV treated animals at day 4 after RSV infection. As with previous experiments, BAL and perfused lung tissue were collected for immune cell quantitation. **A**–**D**, **I**, **J** Total lung leukocytes were determined in BAL and **E**–**H**, **K**, **L** lung tissue following previous OVA and RSV challenge ± anti-IL-33 treatment. Results are representative of three separate experiments. Bars represent the cell count for the indicated populations ± SEM. Mixed ANOVA was used to assess the interaction of anti-IL-33 within the other independent treatments, OVA and RSV. # indicates a significant effect of anti-IL-33 antibody within a treatment groups (P < 0.05). As before * indicates a difference (P < 0.05) between treatment groups

In the last studies we show that neutralizing IL-33 significantly reduces total BALF and lung ILC2 (# P < 0.05 for an anti-IL-33 mAb effect) in only OVA-RSV-treated animals (Fig. 7C, G). Total cellularity was not altered by anti-IL-33 treatments and therefore not the reason for the reduced numbers of ILC2. Along with the BALF and lung ILC2 numbers, eosinophils were decreased in BALF and lung tissue following anti-IL-33 treatment. Interestingly, anti-IL-33 reduced both ILC3 and neutrophils in the BALF and total lung tissue of RSV and OVA-RSV treated animals, but not OVA treated. These results demonstrate a surprising pleiotropic role for IL-33 on both eosinophilic and neutrophilic responses generated following ovalbumin treatment with RSV infection.

## Discussion

Viral infections are the leading cause of hospitalizations and emergency care for asthmatic individuals, and IL-33 is elevated in the serum and sputum of asthmatics following an acute exacerbation [46, 47]. Collectively, studies demonstrated that ovalbumin-induced allergic inflammation is enhanced following RSV infection in an IL-33-dependent manner [48]. The current studies identify unique features of viral infection (RSV), asthma (OVA) and viral induced asthma exacerbation (OVA-RSV) mediated by IL-33. We detected high numbers of eosinophils in the lungs of OVA-RSV-treated animals, and subsequently showed that ILC2 numbers were also elevated in these animals in comparison to single treatment controls. ILC2 and eosinophils were strikingly reduced with anti-IL-33 treatment, which correlated with the reductions in CCL17, CCL22, and IL-5 proteins in the BAL fluid. ILC2 support eosinophil responses and survival in the lung through their production of IL-5 [49–51], and we have shown that ILC2 migrate towards CCL17 and CCL22 in ex vivo transmigration experiments; however, this has not been explored in in vivo experiments utilizing the OVA-RSV model. Because ILC2 produce CCL17 and CCL22 following ex vivo stimulation with IL-33 (Fig. 5), there may be an indirect mechanism by which ILC2 can contribute to allergic inflammation through the recruitment of CCR4 + monocytes and Th2 cells as well [52].

We determined from our neutralization experiments that ILC2 and eosinophils are reliant on IL-33 for recruitment, but two other immune cells, neutrophils and ILC3, were uniquely present in the lungs of RSV- and OVA-RSV-infected mice, and surprisingly both of these populations were significantly reduced with anti-IL-33 treatment at day 4 after RSV infection. Because IL-23 and IL-17 were not significantly reduced with anti-IL-33, we examined additional cytokines systemically to explain the reduced numbers of these cells. Mouse CXCL1, CCL3, CXCL2, CCL2, CCL8 and CCL12 were not different in the serum of RSV and OVA-RSV treated animals following anti-IL-33 treatment. The limitation of our study is that we did not evaluate these additional chemokines in the lungs, and specifically from alveolar macrophages and airway epithelial cells, following anti-IL-33 treatment in the RSV and OVA-RSV treated animals [53–56]. Our future studies will more broadly assess the effects of anti-IL-33 on these prototypical inflammatory markers to determine whether these differences explain the reductions in neutrophils and ILC3. As such, clinical trials are investigating the effectiveness of neutralizing IL-33 in severe asthmatics and those with moderate-to-severe atopic dermatitis. In a phase 2 clinical trial of etokimab (anti-IL-33 mAb), atopic dermatitis patients had significantly reduced neutrophil migration in a CXCR1 dependent manner [57]. CXCR1 and CXCR2 in human neutrophils are well established receptors for the neutrophil chemoattractant, IL-8, and others have shown modulated CXCL1 and chemokine receptor expression by IL-33 in bacterial infections and sepsis in animal models [58, 59]. The results of those studies fit well with our observation of reduced neutrophils following anti-IL-33 treatment. However, while a reduction of IL-33 may be beneficial in allergic diseases, perhaps neutralizing IL-33 disrupts early anti-viral events needed to control the viral infection. Therefore, our future studies will evaluate lung viral titers and pathology in a viral infection model to shed light on potential clinical challenges associated with reducing IL-33.

## Conclusions

This study highlights a significant role of IL-33 in viral-induced asthma exacerbation in the well-known OVA animal model where RSV-A2 was used to amplify mucus and type 2 inflammatory markers. Furthermore, our study shows a significant reduction of neutrophil influx with anti-IL-33 that implies this therapy may be useful in additional chronic pulmonary disorders (COPD, IPF) that are often driven by an unregulated type 3 inflammatory profile involving excess levels of neutrophils.

## Data Availability

All data generated or analyzed during this study are included in this published article.

## Abbreviations

BALF: Bronchoalveolar lavage fluid
CCL17: Chemokine (C–C motif) ligand-17, previously TARC
CCL22: Chemokine (C–C motif) ligand-22, previously MDC
CCR4: Chemokine receptor type-4
CXCL1: C-X-C motif chemokine ligand 1, previously KC or mouse IL-8
CXCR1: C-X-C motif chemokine receptor 1
CXCR2: C-X-C motif chemokine receptor 2
COPD: Chronic obstructive pulmonary disorder
IL-2: Interleukin-2
IL-4: Interleukin-4
IL-5: Interleukin-5
IL-7: Interleukin-7
IL-8: Interleukin-8
IL-17: Interleukin-17
IL-23: Interleukin-23
IL-25: Interleukin-25
IL-33: Interleukin-33
ILC2: Group 2 innate lymphoid cells
ILC3: Group 3 innate lymphoid cells
IPF: Idiopathic pulmonary fibrosis
NKp46: Natural cytotoxicity triggering receptor 1, alternatively CD335 or NCR1
OVA: Chicken egg ovalbumin
TH1: Type 1T helper cell
TH2: Type 2T helper cell
TH17: Type 3T helper cell
TSLP: Thymic stromal lymphopoietin
RSV-A2: Respiratory syncytial virus A2
